# Bayesian adaptive clinical trial designs for respiratory medicine

**DOI:** 10.1111/resp.14337

**Published:** 2022-08-02

**Authors:** Elizabeth G. Ryan, Dominique‐Laurent Couturier, Stephane Heritier

**Affiliations:** ^1^ School of Public Health and Preventive Medicine Monash University Melbourne Victoria Australia; ^2^ Cancer Research UK ‐ Cambridge Institute, University of Cambridge Cambridge UK; ^3^ Medical Research Council Biostatistics Unit University of Cambridge Cambridge UK

**Keywords:** adaptive trial, Bayesian adaptive design, Bayesian methods, clinical trials, interim analysis, monitoring

## Abstract

The use of Bayesian adaptive designs for clinical trials has increased in recent years, particularly during the COVID‐19 pandemic. Bayesian adaptive designs offer a flexible and efficient framework for conducting clinical trials and may provide results that are more useful and natural to interpret for clinicians, compared to traditional approaches. In this review, we provide an introduction to Bayesian adaptive designs and discuss its use in recent clinical trials conducted in respiratory medicine. We illustrate this approach by constructing a Bayesian adaptive design for a multi‐arm trial that compares two non‐invasive ventilation treatments to standard oxygen therapy for patients with acute cardiogenic pulmonary oedema. We highlight the benefits and some of the challenges involved in designing and implementing Bayesian adaptive trials.

## INTRODUCTION

Researchers and funders have recognized the need for efficient clinical trials, and there has been a rapid increase in interest in adaptive designs, particularly during the COVID‐19 pandemic. Clinical trials have commonly used fixed designs, in which data are analysed once all observations have been collected. In such designs, the sample size per arm is typically defined before data collection to achieve a particular level of power given the chosen type I error rate and assumed control and treatment arm distributions. Therefore, they keep key design components constant during the trial and do not make use of accumulating data on the primary outcome as the trial progresses. Often favoured for clinical trials for their simplicity and familiarity, fixed designs can be inefficient, especially if the treatment effect is much larger or smaller than originally anticipated.

Adaptive designs can offer increased flexibility and efficiency for clinical trials by using results from accumulated trial data analysed at scheduled interim looks to alter key design components according to pre‐specified rules. Adaptive designs can help overcome some of the uncertainties about the initial trial design parameter assumptions. Potential adaptations may include: sample size re‐estimation, stopping arms or the trial early for efficacy or futility/lack‐of‐benefit, increasing allocations to more promising treatments (response adaptive randomization), adding new treatments and/or refining recruitment to patients most‐likely to benefit from the intervention (enrichment). Multiple types of adaptations may be used within a trial. Adaptations should be planned a priori to maintain trial integrity and validity.

Adaptive trials are often considered to be more ethical than fixed design trials since they often require fewer patients, potentially saving time and money and/or allocate more patients to better‐performing treatments. Adaptive designs may be used in all clinical trial phases, and may be used to seamlessly transition between consecutive phases. For a general overview on adaptive designs, we recommend Pallman et al.^1^

### Frequentist or Bayesian methods?

Adaptive designs can be performed both within the frequentist and Bayesian statistical frameworks. These two approaches differ in how they define probabilities, how the (unknown) model parameters are treated, how models are formulated and estimated, and how results are interpreted. Frequentist methods, also known as ‘classical’ or ‘traditional’ statistical methods, typically rely on null hypothesis testing involving calculation of test statistics, along with *p*‐values and confidence intervals. Due to their historical dominance frequentist methods are more widely known and practised, especially by clinicians. However, there has been an increasing awareness of the problems associated with these methods (such as misuse, misinterpretation of the *p*‐value and over‐reliance on null hypothesis testing)^2^ and there have been calls to move away from such a paradigm.

Bayesian methods provide an alternative statistical framework and use probability distributions to represent uncertainty of the model parameter estimates (e.g., treatment effect). Bayesian methods provide a formal approach to update parameter estimates as new data are observed, and thus, are an ideal framework for performing interim analyses using accumulating information. Therefore, the use of Bayesian methods in designing and analysing clinical trials has become more prominent and they are naturally better‐suited to performing adaptations compared to frequentist methods.

Bayesian methods incorporate the investigator's initial belief about the unknown parameters of interest (e.g., treatment effect) by specifying *prior distributions* for these parameters. Priors are probability distributions set up before collecting data that account for uncertainty in the parameter estimates. These represent a major difference to frequentist analyses, which assume the parameters to be fixed but unknown in value. Information from previous studies and/or clinical expert opinion can be incorporated into the prior distribution. *Non‐informative* priors can be used as default priors if there are a lack of reliable previous studies or to avoid introducing external information into the analysis and numerically mimic a frequentist analysis. Spiegelhalter et al.^3^ and Berry et al.^4^ provide some discussion around prior distributions for clinical trials.

Once data are collected, new information becomes available and is summarized by another distribution—the *likelihood*. Using Bayes' theorem, the prior is updated by combining it with the S2 likelihood function to become a *posterior distribution*. An example of this updating process is given in Figure S1 / Appendix S1 in the Supporting Information. Decisions and inference can then be made using the posterior which summarizes all information available at that point in time. Bayesian analyses typically report posterior means or medians as parameter (point) estimates, along with credible intervals (in place of confidence intervals) to provide the range of values for the parameters with a certain level of posterior probability (e.g., 95%). We recommend Kruschke^5^ and McElreath^6^ for some introductory texts on Bayesian statistics.

Here, we provide an introduction to Bayesian adaptive clinical trials and discuss some commonly‐used design features/adaptations along with some examples of recent respiratory medicine studies that use these approaches. We focus on later phase trials, particularly multi‐arm trials, since they offer more opportunities for adaptations. We illustrate the approach with a case study, which compares two non‐invasive ventilation strategies to standard oxygen therapy for patients with acute cardiogenic pulmonary oedema. We conclude with a discussion of the benefits and some of the challenges involved in designing and implementing Bayesian adaptive trials.

## BAYESIAN MONITORING OF A TWO‐ARM TRIAL

A frequentist approach may base monitoring of a two‐arm trial (we consider the two‐arm case for simplicity) on a z‐test statistic calculated at each interim analysis, which is compared to a boundary that is derived from a particular mathematical function that is pre‐specified. If the boundary is crossed by the test statistic then the trial may be terminated. Some commonly‐used boundaries include Haybittle‐Peto, O'Brien‐Fleming and α‐spending functions.^7^ These methods maintain the overall control of the type I error despite looking at the data several times. See Jennison and Turnbull^7^ for more details on frequentist approaches.

Bayesian monitoring of a two‐arm trial proceeds using the following logic. At each interim analysis, the posterior distribution of the parameter(s) of interest is computed using the current data, and decisions or adaptations are made using posterior probabilities and pre‐specified rules. For instance, posterior distributions can be used to calculate the probability of a certain sized treatment effect given the information currently available. It then becomes natural to make decisions based on a cut‐off of these posterior probabilities. For example, a trial might permit early stopping for success if the posterior probability that the relative risk (RR) is less than one (given the available information) is greater than the threshold *C* = 0.99, denoted by $\mathrm{Pr}\left(\mathrm{RR}<1|\text{data}\right)>C=0.99$. Clinically meaningful differences could be incorporated into the stopping criteria, for example, by imposing a high probability of observing at least a 10% reduction, that is, $\mathrm{Pr}\left(\mathrm{RR}<0.90|\text{data}\right)>0.99$. By the same token, early stopping for futility may be permitted if the posterior probability the RR < 1 is low, say below 0.10. A more aggressive approach can be chosen by adding a clinically meaningful difference to the futility stopping rule. The cut‐off values *C* are decided by the investigator and may vary across the interim analyses. They are often chosen to achieve control of the (one‐sided) overall type I error at the 2.5% level and Bayesian versions of frequentist stopping boundaries can be constructed for some standard scenarios.^8^ Alternatively, the posterior could be used to define *posterior predictive* probabilities about future observations. Posterior predictive probabilities are typically used to predict whether the trial will be successful if it continues to the planned maximum sample size, or to predict trial success based on completing the current participants' follow‐up, and can be used in stopping rules.^9^

An example of a recent two‐arm trial monitored using the Bayesian paradigm is the CLARITY trial^10^ which compares angiotensin receptor blockers plus standard care to standard care alone in reducing COVID‐19 severity among high‐risk patients. The primary outcome is a 7‐point ordinal scale of clinical outcomes measured at 14 days. The CLARITY trial performs interim analyses every 300 patients, beginning at 700 patients being due for the primary outcome measurement, and uses posterior predictive probabilities to determine whether to stop for efficacy or whether recruiting to the maximum planned sample size (*N* = 2200) would be futile.

## EXAMPLES OF ADAPTATIONS/DESIGNS

The general principles stated above can be used in the context of adaptive trials. There are many ways to define what ‘adaptive’ means but we follow here the definition provided by Gallo et al.^11^: ‘An adaptive design is a clinical study design that uses accumulating data to decide how to modify aspects of the study as it continues, without undermining the validity and integrity of the trial.’ We restrict our attention to late phase Bayesian trials as they are thought to be more relevant in the context of adaptive trials for respiratory diseases, and focus on a few important types of adaptations. A more general review of adaptive trials can be found in Curtin and Heritier.^12^

### Bayesian adaptive sample size and multi‐arm multistage designs

Group Sequential Trials (GSTs), usually conducted as two‐arm studies comparing experimental and control arms, are one of the most commonly‐used adaptive designs based on the above definition. Using the monitoring methods described in the Section ‘Bayesian monitoring of a two‐arm trial’, Bayesian GSTs may stop early if the posterior (predictive) probabilities cross the success or futility thresholds at an interim analysis and otherwise proceed until the next analysis. A substantial reduction in (expected) sample size can be obtained in this setting compared to a standard/fixed trial that does not perform such interim analyses. This was shown for instance in Ryan et al.^13^ who constructed alternative Bayesian GST designs for the OSCAR trial^14^ which compared high frequency oscillatory ventilation to conventional positive pressure ventilation for patients with acute respiratory distress syndrome. Whilst Bayesian GSTs offer some gains in efficiency, the adaptations that can be made in two‐arm trials are limited by nature to early efficacy or futility stopping, changes in the number of looks and sample size reassessment.

Bayesian adaptive sample sizes have been employed when there is uncertainty around the distribution of the primary outcome and around the potential treatment effect sizes as they offer more flexibility than their standard frequentist counterparts.^10^, ^15^ They allow for sample size reassessment through Bayesian monitoring during the accrual phase where the trial may stop early for efficacy or futility (using a GST approach^9^, ^10^), or may continue recruiting if an indeterminant result has been obtained after accrual of the initial planned sample size.^15^

Multi‐arm multistage (MAMS) trials are a natural extension to GSTs offering more gains in efficiency by concurrently comparing multiple experimental arms, usually against a common control arm, and may also permit head‐to‐head comparisons. Multi‐arm trials allow researchers to answer multiple questions within a single regulatory framework and study protocol, rather than running a series of two‐arm trials. Bayesian MAMS trials can be run using a similar approach to that described in the Section ‘Bayesian monitoring of a two‐arm trial’ by computing the posterior probability of a particular treatment being superior to the control. Bayesian *comparative effectiveness* trials may also wish to compute the posterior probability of an intervention being the best arm out of the study treatments explored. Posterior predictive probabilities that consider what might happen if the trial were to continue could also be used. Stopping rules are set using the approach discussed in the Section ‘Bayesian monitoring of a two‐arm trial’, and are restricted by the degree of type I error control (pairwise or overall). In addition to early stopping for efficacy or futility, Bayesian MAMS designs may incorporate adaptations that focus on more promising treatments, such as dropping arms for lack‐of‐benefit, early selection of winners or response adaptive randomization.

### Dropping arms

Arm dropping is one of the logistically simpler adaptations that may be performed in Bayesian MAMS trials and may be used to cease recruitment to ineffective arms. In the Bayesian framework, arms could be dropped if there is a low posterior (predictive) probability that the intervention shows an improvement compared to control (any improvement or a clinically meaningful improvement), or if an arm has a low posterior probability of being the best arm.^16^ Planned maximum sample sizes may be reduced if an arm is dropped, or a study may continue with the original planned maximum sample size to enable more patients to be allocated to more promising treatments.

An example of a Bayesian adaptive trial that incorporated arm dropping is the CATALYST study.^17^ This was a proof‐of‐concept trial, which compared namilumab or infliximab to standard care in hospitalized COVID‐19 patients. Interim analyses were performed every 20 evaluable patients recruited per arm using the accumulated primary outcome data (C‐reactive protein [CRP] concentration over time until day 14). The trial could stop early for success if there was at least a 90% posterior probability that an intervention was superior to standard care in reducing CRP concentration, or an intervention arm could be dropped for futility if there was less than a 50% probability of benefit. The infliximab arm was dropped following an interim analysis which showed a probability of benefit of 21%.

### Bayesian response adaptive randomization

Response adaptive randomization (RAR) is an adaptation that may be suitable for multi‐arm trials^18^ and updates the randomization probabilities based on the information accumulated on the (primary) outcome thus far, in a way that allows more participants to be allocated to better performing arms. These adaptations may be done in stages, or may be performed continuously (i.e., after each participant). A range of methods have been proposed for calculating the randomization probabilities for Bayesian RAR^19^, ^20^, ^21^ including the allocations to the control arm.^20^, ^22^ Prior to the first interim analysis equal randomization may be used, which is often referred to as a ‘burn‐in’ period to allow sufficient preliminary data to be collected.

Many consider RAR to be more ethical than fixed randomization since it can potentially treat more patients with more beneficial treatments^18^ and still provide information on treatment efficacy. Nevertheless, the use of RAR is somewhat controversial, especially with regards to time trends potentially creating biased treatment estimates, the additional operational complexities it introduces and it may not be beneficial in some situations.^21^, ^23^, ^24^, ^25^

An example of a Bayesian RAR design is the endTB trial,^26^ which investigates five new treatment regimens and a control for multidrug‐resistant tuberculosis. The primary outcome was treatment success at 73 weeks and so adaptations were made based on preliminary outcomes (culture conversion at 8 weeks and treatment success at 39 weeks) as RAR typically requires relatively short follow‐up periods. At each interim analysis, the randomization probabilities were updated using a power function of the posterior probability that each intervention arm was superior to control given the current data. The allocation to the control arm was protected by defining the control randomization probability such that its allocation was approximately matched with the experimental arm with the highest number of enrolled patients.

### Seamless designs

Seamless designs may be used to transition between consecutive phases of clinical trials without delay and can potentially utilize data collected in both phases. For instance, a seamless phase II/III trial may investigate multiple doses of a drug and determine the most promising dose(s) in the phase II trial and compare the efficacy of these doses to a control in phase III. The phase II stage could also be used to select among different treatments or subpopulations for investigation in the confirmatory phase III stage. The rules for shifting from phase II to III are pre‐specified and such trials may also decide to abandon the intervention and not proceed to phase III.

Frequentist seamless phase II/III designs have been proposed for chronic obstructive pulmonary disease (COPD) patients^27^, ^28^ and Inoue et al.^29^ illustrate a Bayesian approach for a two‐arm study in non‐small‐cell lung cancer. Alternatively, a Bayesian MAMS approach could be used that allows for early selection of promising arms, dropping poorly performing arms or terminating the trial for futility.

## CASE STUDY

As an illustrative example, we consider a case study that is loosely‐based on the Three Interventions in Cardiogenic Pulmonary Oedema (3CPO) trial.^30^ This is a multi‐arm randomized controlled trial comparing standard oxygen therapy to continuous positive‐pressure ventilation (CPAP) or non‐invasive intermittent positive‐pressure ventilation (NIPPV) for patients with acute cardiogenic pulmonary oedema. The primary outcome in this case study is death within 7 days. Although the original trial pooled the two interventions into a single ‘non‐invasive ventilation’ arm in the primary analysis, we will assume the CPAP and NIPPV arms will be analysed separately (three treatment arms). This is a superiority trial and we are interested in comparisons of the interventions with the control; the experimental arms will not be compared (apart from in the RAR algorithm). The trial maximum sample size is defined using a non‐adaptive frequentist power analysis which showed that a sample size of 1500 patients (500 per arm) will have 80% power to detect the difference of interest, a reduction from 16% to 9.5% in 7‐day mortality, with a two‐sided significance level of 2.5% (Bonferroni correction for pairwise comparisons of each intervention to control).

### Design considerations

In addition to ‘usual’ trial design parameters/features, the following aspects should be considered when designing a Bayesian adaptive trial: (i) the types of adaptations that can be made; (ii) the number and timing of the interim analyses; (iii) the decision rules for the interim and final analyses, including allocation ratios if RAR is used; (iv) the decision thresholds/boundaries and (v) and the prior distributions.

Adaptations should be pre‐specified and depend on the trial objectives. Here we would like to identify better performing arms and drop poorly performing ones as quickly as possible, resulting in the following types of adaptations to be considered: early stopping of the trial for efficacy or futility, RAR and arm dropping.

### Interim monitoring

The number and timing of the interim analyses will depend on the planned maximum sample size, time to observe the primary outcome, recruitment rates and logistical constraints involved in performing the interim analyses and implementing the adaptations. In our case study, we chose to start interim monitoring for early stopping of the trial for efficacy or futility once 750 patients have completed their 7‐day follow‐up and every additional 250 patients completing 7‐day follow‐up thereafter, until early trial stopping or recruiting the maximum sample size (*N* = 1500 patients enrolled).

Our Bayesian adaptive designs assume that the adaptations are driven by the primary outcome alone. Specifically, we will allow early stopping of the trial for efficacy if there is a high posterior probability of *either* CPAP or NIPPV being superior to the control, that is,

$$\mathrm{Pr}\left(P_{\text{CPAP}}<P_{\text{Control}}\right)>S_{i}\;\text{or}\;\mathrm{Pr}\left(P_{\text{NIPPV}}<P_{\text{Control}}\right)>S_{i},$$

where $P_{\text{CPAP}}$, $P_{\text{NIPPV}}$ and $P_{\text{Control}}$ are the 7‐day mortality rates for each arm, and $S_{i}$ is the stopping boundary for superiority at the *i*‐th analysis. We use a similar approach to Connor et al.^16^ where the trial stops accrual once *at least one* superior treatment has been identified. Note that in some situations it may be preferable to stop recruiting to an arm once it has demonstrated efficacy over control (early selection) and not to stop accrual for efficacy unless all intervention arms have demonstrated superiority over control.^31^

Early stopping of the trial for futility (lack‐of‐benefit) may occur if there is a low posterior probability of both CPAP *and* NIPPV being superior to the control, that is,

$$\mathrm{Pr}\left(P_{\text{CPAP}}<P_{\text{Control}}\right)<F_{i}\;\text{and}\;\mathrm{Pr}\left(P_{\text{NIPPV}}<P_{\text{Control}}\right)<F_{i},$$

where $F_{i}$ is the stopping boundary for futility at the *i*‐th analysis. This would indicate that there is little evidence that these treatments are superior to the control.

At the final analysis we consider the trial to be successful if the posterior probability that *either* intervention has a lower 7‐day mortality rate than the control arm rate is greater than 0.9875, that is,

$$\mathrm{Pr}\left(P_{\text{CPAP}}<P_{\text{Control}}\right)>0.9875\;\text{or}\;\mathrm{Pr}\left(P_{\text{NIPPV}}<P_{\text{Control}}\right)>0.9875.$$

Table 1 displays the stopping boundaries $S_{i}$ and $F_{i}$, and the final analysis success criterion for our design. These values were tuned via simulations. A discussion on how the stopping boundaries may be chosen is provided in Appendix S2 in the Supporting Information.

**TABLE 1:** Timing of interim analyses and posterior probabilities required to stop for success (*S*) or futility (*F*) at each interim analysis, and final analysis success criteria for the case study

Interim analysis	Number of patients with complete follow‐up	$S_{i}$	$F_{i}$
1^a^	500	NA	NA
2	750	0.9984	0.1003
3	1000	0.9963	0.2591
4	1250	0.9928	0.5411
Final^b^	Max 1500	0.9875	NA

*Note*: $S_{i}$ is the stopping boundary for superiority at the *i*‐th analysis; $F_{i}$ is the stopping boundary for futility at the *i*‐th analysis.

^a^ Only response adaptive randomization (RAR) was performed at the first interim analysis (i.e., no early stopping for efficacy or futility was permitted).

^b^ If the trial did not stop early for efficacy or futility, the final analysis was performed once 1500 patients were recruited and followed up; if the trial stopped early, then the final analysis was performed once the recruited patients completed follow‐up.

Unlike Gotmaker et al.,^31^ who chose to control the pairwise error rate (experimental arm vs. control) at 2.5% under the null hypothesis, our stopping boundary values control the *overall* type I error rate at the 2.5% level whilst preserving high power (>80%) for a meaningful effect size (a reduction from 16% to 9.5% in 7‐day mortality). Note that this Bayesian adaptive design is constructed as a one‐sided superiority study, which is common for this approach.^10^, ^16^

### Response adaptive randomization and arm dropping

Prior to the first interim analysis, equal randomization is used (burn‐in period). We first update the randomization probabilities after the 500th patient has completed their 7‐day follow‐up and then every 250 patients. The randomization probabilities for the intervention arms are updated to be proportional to the posterior probability that the arm is the best intervention arm.^21^ The control allocation probability is matched to the allocation probability of the best intervention arm (i.e., the intervention arm with the highest allocation probability) to ensure there is sufficient power for comparisons with the control.

Enrolment to intervention arms could be suspended if they had a low randomization probability (<0.1), that is, were performing poorly, allowing the remaining intervention arm to receive an increase in allocations. The suspended arm could resume enrolment at subsequent interim analyses if the randomization probability increased above the threshold. This is known as ‘temporary arm dropping’ and is implemented in the simulation package used for this case study when RAR is employed.^32^ Treatment and follow‐up continues for patients in the suspended arm. Permanent arm dropping may be preferred in some situations, where enrolment is ceased to an arm for futility, and the trial may stop for futility if all intervention arms are dropped.

### Prior distribution

A discussion on the choice of prior distribution is provided in Appendix S3 in the Supporting Information. We note here that the choice of the prior is less critical when large samples are used, like in this example, as the information provided by the data (via the likelihood) is dominant in such cases (see also Figure S2 in the Supporting Information).

### Simulation settings

During the design phase, computer simulations are typically conducted under a range of plausible scenarios (e.g., treatment response rates or distributions, treatment effect sizes, stopping rules and recruitment rates) and incorporate the adaptations of interest. Table 2 presents the scenarios of interest for this study.

**TABLE 2:** Effect size scenarios explored for the Bayesian adaptive design case study

Scenario	$P_{\text{Control}}$ (%)	$P_{\text{CPAP}}$ (%)	$P_{\text{NIPPV}}$ (%)
(1) Null	16%	16%	16%
(2) One intervention superior	16%	16%	9.5%
(3) Both interventions superior	16%	9.5%	9.5%
(4) NIPPV > CPAP > Control^a^	16%	12.5%	9.5%
(5) Both interventions have small improvement	16%	12.5%	12.5%
(6) Harm	16%	18%	18%

*Note*: $P_{\text{Control}},P_{\text{CPAP}}$, $\;P_{\text{NIPPV}}$ are the 7‐day mortality rates for each arm; Note that $P_{\text{CPAP}}$ and $P_{\text{NIPPV}}$ could be interchangeable here, depending on whether the clinicians thought that continuous positive‐pressure ventilation (CPAP) or non‐invasive intermittent positive‐pressure ventilation (NIPPV) were more likely to be superior.

^a^ Here ‘>’ means ‘better than’, that is, have lower 7‐day mortality.

The frequentist properties of the design, known as *operating characteristics*, are studied by simulating the trial a large number of times (e.g., 10,000) for each scenario. Different quantities are typically recorded using the simulation results (power, type I error, average sample size, proportion of simulations which stopped early and average allocations to each arm) for each scenario, as analytical solutions are usually not available. Features of the design can be updated and simulations rerun if results are not as expected (e.g., high type I error). The simulations of our trial design are performed using the commercial software FACTS.^32^

### Results

**FIGURE 1:**
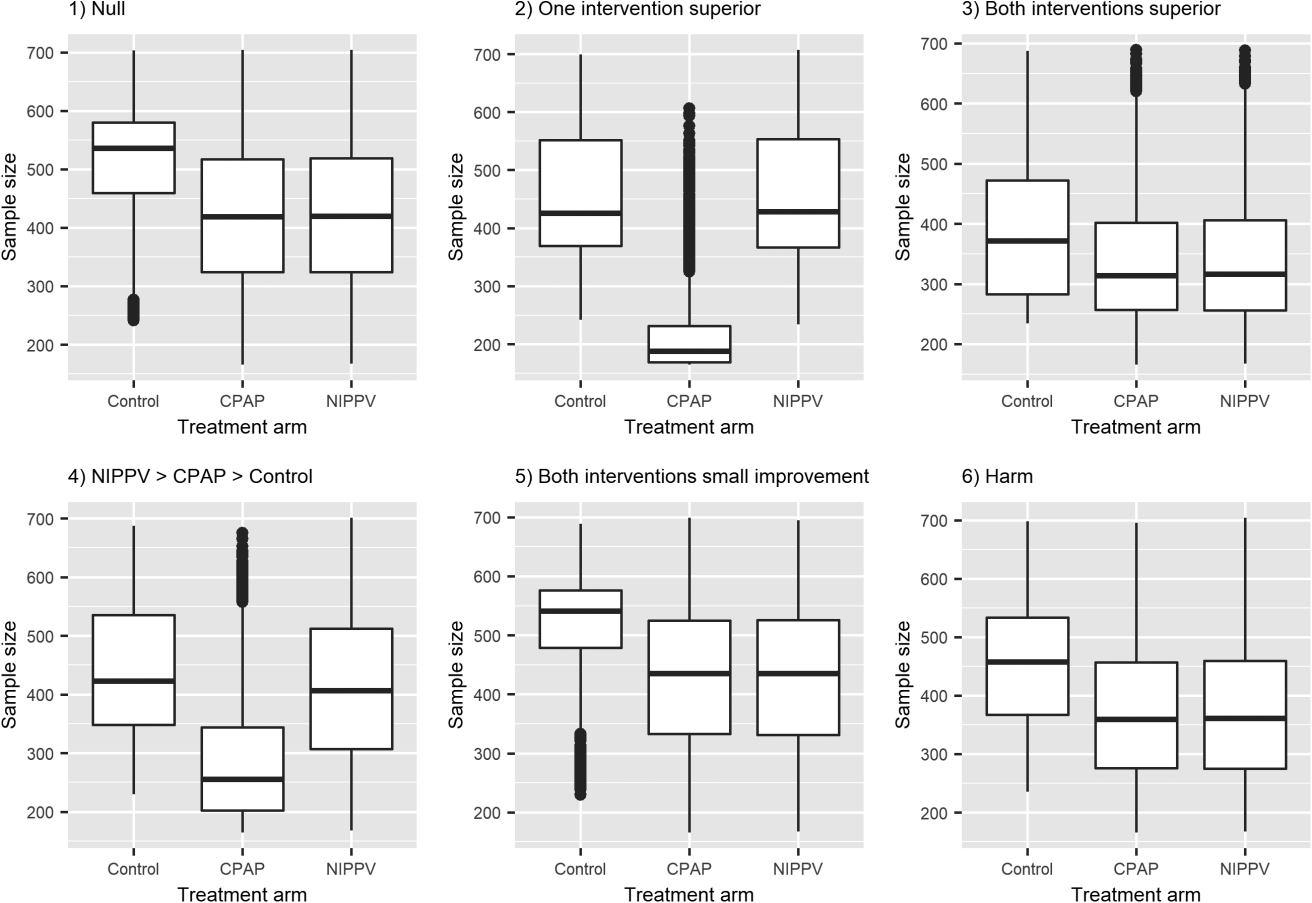
Boxplots showing the distribution of allocations (number of patients) for each treatment arm across the 10,000 simulated trials for each scenario (represented in separate plots) for the Bayesian adaptive design case study

Table 3 presents the operating characteristics for our Bayesian adaptive design. It has an overall (one‐sided) type I error close to our target of 0.025 (scenario 1) and a probability of at least 0.86 to declare the trial to be successful when at least one intervention arm has a clinically‐relevant effect. For the scenarios where only one arm has a clinically‐relevant effect (scenarios 2 and 4), we obtain a probability of at least 0.82 of declaring that arm to be superior to control. However, when both arms have a clinically‐relevant effect (scenario 3), the probability of declaring each intervention superior to control (individually) decreases due to the decreased average sample size induced by the early stopping for efficacy. The trial is often stopped for futility (73.2% of simulations) when the interventions are harmful (scenario 6). Compared to the fixed design (*N* = 1500), the average sample sizes are reduced by approximately 130–500 patients in the scenarios explored, with the biggest savings occurring in the presence of a clinically‐relevant benefit. RAR allocates more patients to superior interventions but may also result in more early stopping for efficacy. Similar numbers of patients are allocated to arms that have the same treatment effect. This can be seen in Figure 1 displaying the distribution of the sample sizes for each arm for each scenario.

**TABLE 3:** Operating characteristics for the Bayesian adaptive design case study

Scenario	Proportion of simulations CPAP declared superior to control	Proportion of simulations NIPPV declared superior to control	Proportion of simulations where at least one intervention is declared superior to control^a^	Proportion of simulations stopped early for success	Proportion of simulations stopped early for futility	Average total sample size (SD)	Average allocations, number of patients (SD)		
							Control	CPAP	NIPPV
(1) Null	0.0133	0.0144	*0.0258*	0.0155	0.3925	1356 (202)	515 (92)	421 (123)	421 (123)
(2) One intervention superior	0.0108	0.8897	0.8901	0.7470	0.0018	1126 (273)	458 (126)	211 (59)	458 (127)
(3) Both interventions superior	0.6710	0.6745	0.9260	0.8193	0.0004	1064 (269)	391 (106)	336 (105)	337 (106)
(4) NIPPV > CPAP > Control	0.1700	0.8274	0.8604	0.7166	0.0019	1140 (278)	436 (115)	285 (102)	421 (115)
(5) Both interventions have small improvement	0.2559	0.2519	0.4314	0.2821	0.0267	1370 (221)	517 (93)	427 (122)	427 (123)
(6) Harm	0.0005	0.0017	0.0021	0.0012	0.7319	1197 (242)	451 (107)	373 (117)	374 (117)

*Note*: These results are based on 10,000 simulated trials for each scenario. We assume a mean recruitment rate of 6.5 patients/week and that it took 6 months to reach that rate. It was assumed there would be no dropouts.

Abbreviations: CPAP, continuous positive‐pressure ventilation; NIPPV, non‐invasive intermittent positive‐pressure ventilation.

^a^ Proportion of simulated trials that declared the trial to be ‘successful’, that is, at least one arm superior to the control at the final analysis (includes trials that stopped early and those that recruited to the maximum sample size). The simulated type I error is italicized.

## MORE COMPLEX DESIGNS—PLATFORM TRIALS

Platform trials evaluate multiple treatments (or treatment combinations) simultaneously in one or more patient subgroups for a disease or condition and operate under a *single master protocol*. Adaptive platform trials also incorporate adaptive features and can be perpetual or open in that the number of treatment arms is not fixed and treatment arms may be added or removed during the trial, according to predefined criteria.^33^ The control arm may also be updated with a new superior treatment once it has been approved, and other flexible features such as RAR or enrichment may be incorporated.

Adaptive platform trials typically use multifactorial designs where patients can be randomized to one or more different domains of treatment (such as antibiotics, antivirals, corticosteroids and mechanical ventilation strategies).^34^ This allows for simultaneous testing of different treatment strategies and combinations of treatments. As such, they may be viewed as an extension of MAMS designs (where a single domain is typically studied). Different inclusion criteria may be applied for each domain, and certain patient populations may be targeted for the testing of particular treatment arms. New domains of treatment or new disease subgroups may also be added to the adaptive platform.^34^

Adaptive platform trials utilize protocol amendments and appendices to the master protocol, rather than designing a new trial for each comparison, for each new treatment that becomes available, or for new domains or subgroups that are to be explored. Thus, platform trials are highly modular. These trials may enable resources to be shared between multiple sponsors and can find effective treatments more efficiently compared to traditional randomized controlled trials.^35^

The Bayesian approach is well‐suited for implementing the more complex features of adaptive platform trials, such as RAR, hierarchical modelling (which allows borrowing of information about treatment effects across subgroups), longitudinal modelling and assessment of treatment combinations across subgroups.^35^ One of the most well‐known Bayesian adaptive platform trials is the REMAP‐CAP study^34^, ^36^ which was originally designed for adult patients with severe community‐acquired pneumonia but also included infrastructure for pandemic respiratory infections in its design (‘Pandemic Appendix’). REMAP‐CAP is a perpetual adaptive platform trial that has included at least 55 interventions in 16 domains (as of April 2022),^36^ and has been used to investigate many treatments for COVID‐19 and avoided the need to set up multiple new trials. Bayesian adaptive platform trials are also being conducted in other respiratory diseases, such as cystic fibrosis^37^ and influenza‐like illness.^38^ Platform trials can provide many opportunities for faster therapy approval and the U.S. Food and Drug Administration (FDA) has recently released a guidance document on Master Protocols.^39^

## WHEN TO USE/NOT USE (BAYESIAN) ADAPTIVE DESIGNS

Implementation of adaptive designs requires much more statistical planning for both the study design and analysis, and a more complex administrative infrastructure compared to fixed/traditional designs. Additionally, planning of a Bayesian adaptive trial can be more complex than for frequentist designs with similar adaptive features and there are generally not many off‐the‐shelf designs available. Custom code or software are generally required to determine the operating characteristics of Bayesian adaptive designs, some of which may be computationally intensive. We discuss some of the software available for Bayesian adaptive designs in Appendix S4 in the Supporting Information.

Although (Bayesian) adaptive designs can be more efficient than traditional designs in many scenarios, they can be more complicated to implement and there are some situations where they might not be worthwhile. Recruitment rates and duration of follow‐up play important roles in adaptive designs. Studies with fast recruitment and relatively long periods to observe the primary outcome may have little information available at interim analyses, or may finish recruitment before any adaptations can be implemented. Intermediate outcomes may be required for trials with long‐term primary outcomes to enable interim decision‐making, provided they are informative and strongly correlated with the primary outcome and are observed fairly quickly.^26^, ^40^ One should consider the availability of resources to deliver a potential increase in the allocations to certain treatment arms (via RAR or arm dropping), as this may not be feasible for some types of treatments. To perform the interim analyses, data must be collected, entered, cleaned and analysed in a timely fashion and to a high standard. To implement RAR or suspend recruitment to a treatment arm, one must be able to update the randomization system rapidly and with ease.

Other operational barriers (including additional costs/resources), potential presence of secular trends, trial complexity, the importance of secondary/long‐term outcomes and potential subgroup effects and personnel expertise should also be considered when deciding whether to implement Bayesian adaptive designs for a particular trial to achieve the trial's objective.^40^ Due to their complexity, platform trials require additional design considerations, which have been discussed in recent overviews.^33^, ^41^ Ideally, Bayesian adaptive designs should be generated by biostatisticians with expertise in these designs.

## DISCUSSION

We have discussed some of the common adaptations that may be implemented in Bayesian adaptive trials and provided several examples of recent respiratory medicine studies that have used these approaches. The potential adaptations are not limited to the examples provided here and other adaptations/designs may be implemented, such as: early selection, adaptive enrichment or dose‐escalation. Through our case study, we have provided a brief outline of the process involved in constructing a Bayesian adaptive design; extensions to this process are required for more complex designs, such as adaptive platform trials. This article is intended as a starting point for clinicians interested in Bayesian adaptive designs and we encourage readers to seek a deeper understanding by reading some of the cited materials.

Bayesian adaptive designs provide a flexible way of designing clinical trials, particularly those with complex features. We have shown through our case study how Bayesian adaptive designs can provide gains in efficiency by potentially reducing the sample size (compared to a standard frequentist approach) and increasing allocations to more promising treatment arms, whilst maintaining high power. The decision rules that we have implemented here may be more interpretable to clinicians, rather than those based on test statistics (used in frequentist designs).

The case study that we have presented is for illustrative purposes only. All Bayesian adaptive designs are situation‐specific and the adaptations and decision rules should be tailored to the trial's objectives. The impact of each adaptive feature on the design's operating characteristics should be thoroughly investigated pre‐trial.

## AUTHOR CONTRIBUTION

Elizabeth G. Ryan constructed the Bayesian adaptive design for the case study, with feedback from Stephane Heritier and Dominique‐Laurent Couturier, ran the simulations for the design, generated the figures, and drafted the manuscript; Dominique‐Laurent Couturier validated the output from the simulations; Stephane Heritier directed the research; all authors discussed and commented on the manuscript. All authors read and approved the final manuscript.

## CONFLICT OF INTEREST

None declared.

## Supporting information


**Supporting information**.
