# Supplementary material for: Bayesian adaptive clinical trial designs for respiratory medicine
Source: Respirology. 2022 Aug 2;27(10):834–43. doi: 10.1111/resp.14337 (PMC9544135; doi:10.1111/resp.14337)
Supplement: Supplementary file 1 — Supporting information. [file RESP-27-834-s001.docx]

**Supporting Information**

**Appendix S1- UPDATING PRIOR TO POSTERIOR DISTRIBUTION**

The process of updating the prior information with new data to form the posterior distribution (via Bayes’ theorem) is demonstrated in Figure S1. Here we use a simple example of estimating an event rate or proportion, say, mortality at a particular time point. Figure S1 also demonstrates the influence of a prior on the posterior when the sample size is relatively small (panel A, N=100) and large (panel B, N=1000). Gates et al.^1^ provide a demonstration of the influence of priors of different levels of informativeness (non-informative, moderately informative, informative) when combined with different sized datasets, on the posterior distribution.

We use the posterior distribution to make inferences on the parameters of interest. For example, using the results from Figure S1 we could calculate the probability that the mortality rate (p) is < 0.5 or > 0.3.


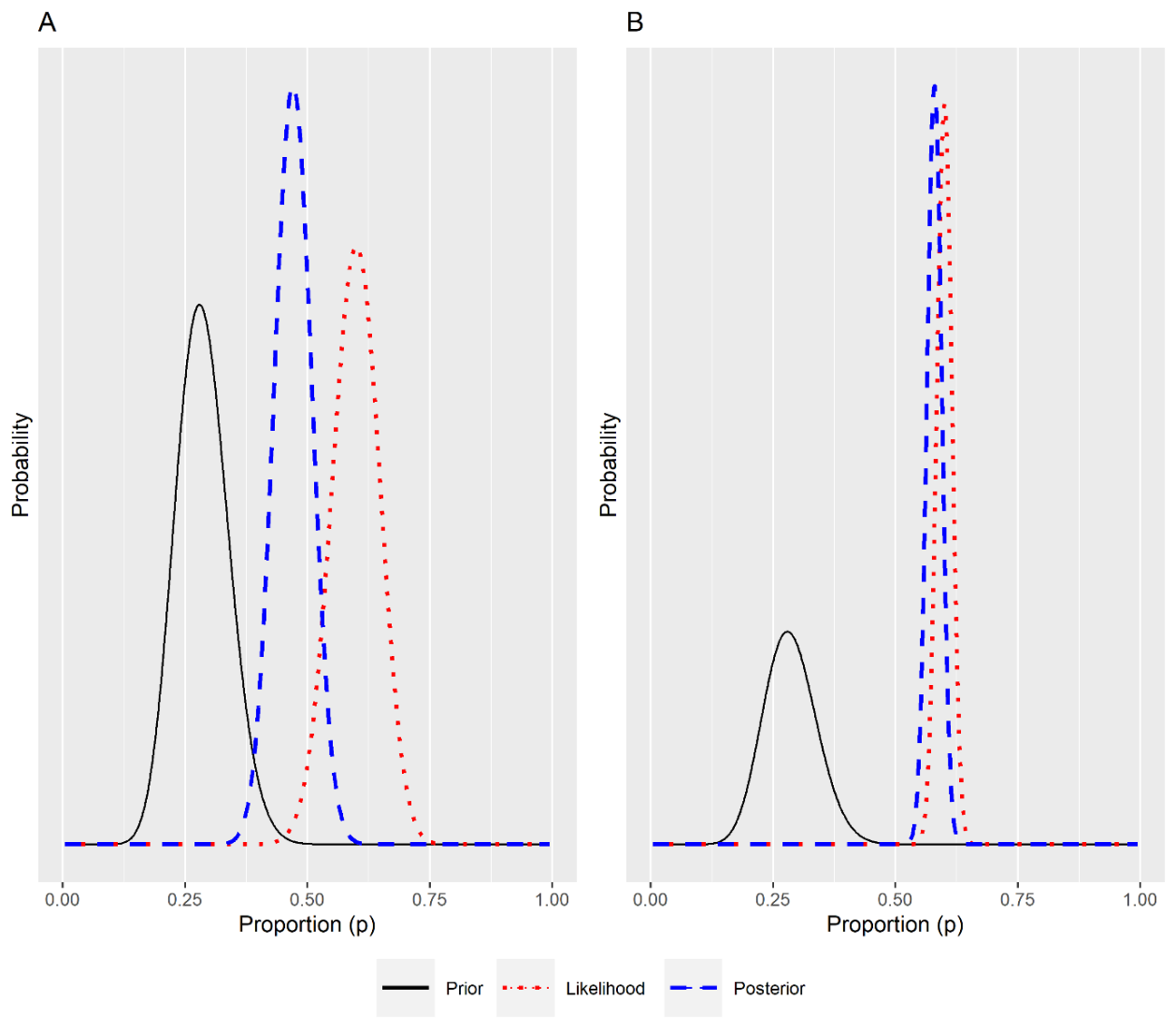


**Figure S1.** An example of prior, likelihood and posterior distributions for a proportion (e.g., proportion of patients that died within 7 days of randomisation) demonstrating the updating of the previous information with the current data by using a Bayesian analysis. An informative prior is used and combined with A) N=100, B) N=1000 observations, demonstrating that the prior is less influential with larger sample sizes (information provided by the data, via the likelihood function, dominates). In this example all the distributions are Beta distributions.

In the context of a standard two-arm randomised controlled trial, one could be interested in the difference in mortality rates or a relative risk and could similarly base decisions on the posterior of these treatment effect measures. Gates et al.^1^ illustrate some of the quantities of interest that may be derived from such a posterior distribution.

**Appendix S2- CHOICE OF STOPPING BOUNDARIES FOR CASE STUDY**

A decreasing structure for $S_{i}$ (stopping boundary for superiority at the *i*-th analysis) and an increasing for $F_{i}$ (stopping boundary for futility at the *i*-th analysis) are typically chosen, the rationale being that more stringent criteria are preferred early on when fewer patients have data available and that the level of evidence required to make decisions could be relaxed when the number of observations increases. This is also in line with frequentist methods; for instance, the O’Brien-Fleming efficacy stopping rule requires a strict cut-off early on and is progressively relaxed. Rather than using z-test statistic values, the Bayesian approach expresses the stopping boundaries on the posterior probability scale. Choice of the stopping boundaries depends on the investigator. Similar values to those employed in previous studies can provide a starting point and then their values are “tuned” to give good operating characteristics.^2-4^ Another possibility is to choose $S_{i}$ and $F_{i}$ as functions of the “information fraction” present in the data, i.e*.*, *n(interim)/n(max),* and choosing the related parameters to achieve certain targets in terms of type I error (or power)^5,6^; this is the approach used in our case study.

**Appendix S3- PRIOR DISTRIBUTIONS FOR CASE STUDY**

The form of the prior distribution will depend on the model being used for the data. It can be constructed using domain knowledge based on expert clinical opinion or information from previous studies, or can be constructed to be minimally informative and allow the observed data to drive the inference. The form of the prior distribution may be limited by the statistical package or software being used.

For this case study we use normal distributions for the priors for the log-odds of the 7-day mortality rate for each treatment arm. The simulation software that was used (FACTS^7^) requires priors to be specified in this format for binary outcomes; other parameterisations are possible in different software. The chosen priors produce a median 7-day mortality rate of 16% and a 95% credible interval of 6.7-33.6% (Figure S3). The same independent priors are used for each arm, and no assumptions are made regarding superiority of an arm. We did not use a flat prior (which would imply all 7-day mortality rates were equally likely) as we thought that high 7-day mortality rates would be very unlikely. Since we have a fairly large sample size, the choice of the scale of the prior is not as important as with small samples where there is less information being contributed by the data (via the likelihood).


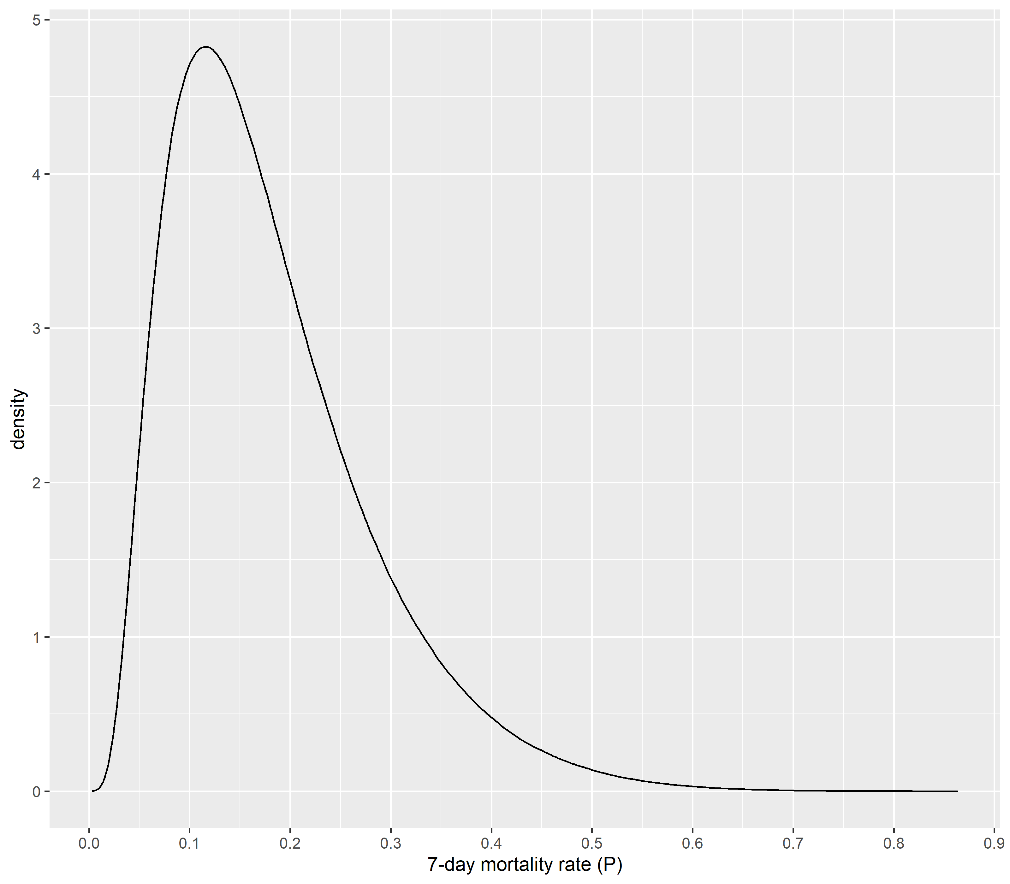


**Figure S2.** Prior distribution for the primary outcome, 7-day mortality rate, that was used for each treatment arm in the simulations of the case study

**Appendix S4- SOFTWARE FOR BAYESIAN ADAPTIVE DESIGNS**

One of the barriers in implementing Bayesian adaptive designs is the lack of easily accessible, user-friendly software to perform the simulations required to study the designs’ operating characteristics and perform trial analysis. Bayesian adaptive designs require a large amount of pretrial work to be performed via simulations which examine the consequences of the adaptations and decision rules under various plausible scenarios.

Several packages have been developed using the R software^8^ for simulating Bayesian group sequential designs,^9-11^ but these do not offer much flexibility in the scenarios and design settings that can be explored. Currently, simulation and analysis of Bayesian adaptive designs generally requires either constructing bespoke code in a computer programming language (such as R, SAS, FORTRAN) or using standalone simulator software (such as FACTS^7^ or HECT^12^). The advantage of the latter is that these simulators do not require the user to perform any programming and perform the trial simulations relatively quickly, but do have limitations in the designs that can be explored. A recent review by Grayling and Wheeler^13^ found that the R software^8^ was the most commonly used for writing the statistical programs for Bayesian adaptive designs. Meyer et al.^14^ provide an overview of the software potentially available for designing platform trials, and include options for Bayesian designs (e.g., OCTOPUS^15^) which are undergoing development.

**REFERENCES**

1. Gates S, Brock K, Ryan EG. Bayesian statistical methods and their application to resuscitation trials. *Resuscitation*. 2020; **149**:60-64.
2. Broglio KR, Connor JT, Berry SM. Not too big, not too small: a goldilocks approach to sample size selection. *J. Biopharm. Stat.* 2014; **24:** 685-705.
3. Ryan EG, Bruce J, Metcalfe AJ, Stallard N, Lamb SE, Viele K, Young D, Gates S*.* Using Bayesian adaptive designs to improve phase III trials: a respiratory care example. *BMC Med. Res. Methodol.* 2019; **19:**99.
4. [Connor JT](https://www.ncbi.nlm.nih.gov/pubmed/?term=Connor%20JT%5BAuthor%5D&cauthor=true&cauthor_uid=23983160), [Luce BR](https://www.ncbi.nlm.nih.gov/pubmed/?term=Luce%20BR%5BAuthor%5D&cauthor=true&cauthor_uid=23983160), [Broglio KR](https://www.ncbi.nlm.nih.gov/pubmed/?term=Broglio%20KR%5BAuthor%5D&cauthor=true&cauthor_uid=23983160), [Ishak KJ](https://www.ncbi.nlm.nih.gov/pubmed/?term=Ishak%20KJ%5BAuthor%5D&cauthor=true&cauthor_uid=23983160), [Mullins CD](https://www.ncbi.nlm.nih.gov/pubmed/?term=Mullins%20CD%5BAuthor%5D&cauthor=true&cauthor_uid=23983160), [Vanness DJ](https://www.ncbi.nlm.nih.gov/pubmed/?term=Vanness%20DJ%5BAuthor%5D&cauthor=true&cauthor_uid=23983160), Fleurence R, Saunders E, Davis BR. Do Bayesian adaptive trials offer advantages for comparative effectiveness research? Protocol for the RE-ADAPT study. *Clin. Trials.* 2013; **10**: 807-827.
5. Cellamare M, Ventz S, Baudin E, Mitnick CD, Trippa L. A Bayesian response-adaptive trial in tuberculosis: The endTB trial. *Clin. Trials*. 2017; **14**:17-28.
6. Gotmaker R, Barrington MJ, Reynolds J*,* Trippa L, Heritier S*.* Bayesian adaptive design: the future for regional anesthesia trials? *Reg. Anesth. Pain Med.*2019; **44:**617-622.
7. Fixed and Adaptive Clinical Trial Simulator (FACTS). Version 6.4. 2021. Berry Consultants, Austin TX.
8. R Core Team, 2021. R: A language and environment for statistical computing. R Foundation for Statistical Computing, Vienna, Austria. <https://www.R-project.org/>.
9. Gerber F, Gsponer T. gsbDesign: An R Package for Evaluating the Operating Characteristics of a Group Sequential Bayesian Design. *J. Stat. Softw.* 2016; **69:** 1–23.
10. Eggleston BS, Ibrahim JG, McNeil B, Catellier D. BayesCTDesign: An R Package for Bayesian Trial Design Using Historical Control Data. *J. Stat. Softw*. 2021; **100:** 1-51.
11. Hickey GL, Wan Y, Chandereng T, 2021. goldilocks: Goldilocks Adaptive Trial Designs for Time-to-Event Endpoints. R package version 0.3.0. <https://CRAN.R-project.org/package=goldilocks> Accessed: 25 March 2022
12. Thorlund K, Golchi S, Haggstrom J, Mills E. Highly Efficient Clinical Trials Simulator (HECT): Software application for planning and simulating platform adaptive trials. *Gates Open Res*. 2019; **3**:780.
13. Grayling MJ, Wheeler GM. A review of available software for adaptive clinical trial design. *Clin. Trials*. 2020; **17**:323-331.
14. Meyer EL, Mesenbrink P, Mielke T, Parke T, Evans D, König F*.* Systematic review of available software for multi-arm multi-stage and platform clinical trial design. *Trials*. 2021; **22:**183.
15. Wathen JK, 2020. Octopus - optimize clinical trials on platforms using simulation. <https://github.com/kwathen/OCTOPUS> Accessed: 25 March 2022.
